# Editorial: Special Issue: “*Legionella pneumophila*: A Microorganism with a Thousand Faces”

**DOI:** 10.3390/microorganisms11102392

**Published:** 2023-09-25

**Authors:** Teresa Fasciana, Mario Palermo, Ignazio Arrigo, Maria Rita Tricoli, Orazia Diquattro, Anna Giammanco

**Affiliations:** 1 Department of Health Promotion, Mother and Child Care, Internal Medicine and Medical Specialities, University of Palermo, 90127 Palermo, Italy; teresa.fasciana@virgilio.it; 2 Legionella Reference Laboratory, University of Palermo, 90127 Palermo, Italy; ignazio.arrigo90@gmail.com (I.A.); mariaritatricoli@gmail.com (M.R.T.); 3 Sicilian Health Department, Public Health and Environmental Risks Service, 90127 Palermo, Italy; mario.palermo1955@gmail.com; 4 Laboratory of Microbiology, A. O. Ospedali Riuniti “Villa Sofia-Cervello”, 90100 Palermo, Italy; orazia.diquattro@villasofia.it

*Legionella pneumophila* is a microorganism that is able to contaminate the freshwater environment and, consequently, human-made water systems. Its pathogenicity is related to the host’s ability to exhibit pathogenic factors that influence the intracellular growth of *L. pneumophila* in macrophages as well as in protozoa, which is also mediated by biofilm production [1,2]. Legionellosis outbreaks are mainly related to the presence of biofilms, which can be prevented by reducing water system contamination [3]. Currently, considering that legionellosis is more commonly associated with the presence of other *Legionella* species inside the water supply system, the opportunity to seek out these others as well as *L. pneumophila* is considered relevant. The main focus of this Special Issue is analyzing epidemiological data, mechanisms and host factors involved in Legionnaires’ disease and expanding information on the mechanisms of biofilm formation and concerning the activity of old and new anti-biofilm products, as well as the implications for co-infection.

As an innovative system for avoiding *Legionella* biofilm formation in water systems, a type of coating based on the use of a sulfonated pentablock copolymer (s-PBC, commercially named Nexar™) covered with a more hydrophilic s-PBC, an acid, and a negatively charged surface (Nexar™-modified) is proposed. The Nexar™-modified system’s effect caused by the acid and the negative surface of s-PBC is able to preclude both bacteria adhesion to the filter and its proliferation via the acidification of water strictly in contact with the filter surface. In fact, the polymer was shown to be able to block *Legionella* on the filter surface and prevent the bacteria from persisting in water, leading to microbial physiological inhibition, and thus it can be used to reduce planktonic and sessile (biofilm) *Legionella* cells [4,5,6,7,8].

Biofilm production is also observed in *L. pneumophila* sgr 2–15 strains, which are particularly resistant to water disinfection processes, meaning that they are difficult to eradicate from the environment as well from hospital water supplies. Their presence and the increase in *Legionella* infections are associated with other *Legionella species* such as *L. anisa*, indicating the role of monitoring and surveillance in preventing *Legionella* spp. infections, particularly emphasizing the importance of checking the conditions of hospital facilities, to enable efficient prevention [9,10,11].

*Legionella* present in biofilms and sediment may be mobilized by boiler flushing, water demand (informally called stagnation), as well as by the temperature and flow, considered as other *Legionella* spp. growth factors [12,13,14].

At present, different factors have been associated with a variable increase in *Legionella* spp. response due to building occupancy patterns and flushing [15]. *Legionella* increases were also confirmed to be related to flow rate and a reduction in boiler temperature.

Reduced water demand associated with low building occupancy does not always cause *Legionella* growth, even when the building has been colonized by *Legionella* previously; however, some flushing practices may temporarily increase *Legionella* occurrence. It has been hypothesized that rapid boiler turnover, high shear sloughing of biofilms associated with flushing, using many outlets simultaneously, and rapid nutrient influx could contribute to increased Legionella occurrence.

Another factor influencing the increase in *Legionella* spp. is the hydraulic retention time (water age) in building water systems, namely the water age of the distribution system, through the local plumbing as well as building management [16,17]. This increase has been associated with the presence of the five most frequent *Legionella* species in the groundwater system, as well in taps and cooling towers. *Legionella* spp. were found in 100% of water samples; more specifically, *L.pneumophila* was found in 57% of the water samples, followed by *L. bozemanii*, *L. longbeachae*, *L. micdadei*, and *L. anisa* [18]. In addition, in whole water supply systems, it is important to consider other relevant species and subsequently to control the growth of the five most pathogenic *Legionella* species in the built water environment.

More and more frequently, *Legionella* has been identified in relevant sources extending to environmental habitats that actually are not commonly linked to human disease [19]. The documentation of all possible reservoirs for *Legionella* spp. is considered relevant to acquire data on the presence of *Legionella* in environments less often examined, mainly to develop procedures to limit transmission and diffusion beyond cooling towers and household plumbing.

It is widely accepted that the ability of *L. pneumophila* to thrive within the biofilm of household plumbing systems is based on utilizing protozoan hosts for protection from environmental stressors and to increase its growth rate and the bacteria’s infectivity into human host cells. The genes that regulate protein secretion and ultimately the phylogenic characteristics of effective replication inside and outside the host cells are used to model the lifecycle of the bacteria. The information regarding *L. pneumophila*’s growth within and without the host cell, related to the genes which influence these processes, which can be used to compute how oxidative stress can downregulate those genes, is summarized in [20].

The implications of *Legionella* in co-infection were especially on interest during the COVID-19 pandemic period. The observations indicate that the occurrence of co-infections of SARS-CoV-2 and *Legionella* may correspond to a comparatively rare but not irrelevant case, outlined by a severe prognosis [21]. The risk of co-infection has been measured, particularly focusing on risk factors, and two main outcomes—ICU admission and the case fatality ratio (CFR)—have been noted [22].

In Italy, the prevalence of legionellosis is diverse and related to geographic variability. The overall analysis of the causes and the report regarding the relative differences in incidence between Italian regions emphasize that these differences cannot to be exclusively attributed to the possible influence of environmental factors (demographic factors as age and sex distribution of the population); instead, they may be related to climate and demographics factors (i.e., age and sex distribution of the population), the choice of different diagnostic tests (PCR, culture, or urine antigen test), the efficiency of the surveillance systems, variations in climate, geographic risk factors, and notification rates, which are higher in northern regions compared to the southern ones and the main islands [23]. In regions with persistently low notification rates, ad hoc studies are recommended to assess the reasons for underestimation.

In conclusion, this Special Issue combines manuscripts with exclusive purposes: to evaluate the microbial factors of *Legionella* spp. implicated in pathogenesis through specific virulence factors such as biofilm formation, as well as to advise environmental surveillance to reduce microorganism circulation.
